# Exploring the role of ionic liquids to tune the polymorphic outcome of organic compounds†
†Electronic supplementary information (ESI) available: CCDC 1577981. For ESI and crystallographic data in CIF or other electronic format see DOI: 10.1039/c7sc04353h


**DOI:** 10.1039/c7sc04353h

**Published:** 2017-12-22

**Authors:** Qingying Zeng, Arijit Mukherjee, Peter Müller, Robin D. Rogers, Allan S. Myerson

**Affiliations:** a Novartis-MIT Center for Continuous Manufacturing , Department of Chemical Engineering , Massachusetts Institute of Technology , 77 Massachusetts Avenue , Cambridge , Massachusetts 02139 , USA . Email: myerson@mit.edu; b Department of Chemistry , McGill University , 801 Sherbrooke St. W. , Montreal , QC H3A 0B8 , Canada; c Department of Chemistry , Massachusetts Institute of Technology , 6-331, 77 Massachusetts Avenue , Cambridge , Massachusetts 02139 , USA

## Abstract

While molecular solvents are commonly used in the screening of polymorphs, the choices are often restricted. Ionic liquids (ILs) – also referred as *designer solvents* – have immense possibility in this regard because of their wide flexibility of tunability. More importantly, the interactions among the IL components are completely unique compared to those present in the molecular solvents. In this context, we have chosen tetrolic acid (TA) and isonicotinamide (INA), which showed solution-structure link in molecular solvents in the past, as probes to investigate the role of imidazolium based ionic liquids in the polymorphism of these two systems and whether the different solute–solvent interactions in ILs affect the polymorphic outcome. It is observed that the selected imidazolium-based ILs, with varying anion basicity have influenced the crystallization outcome by the interaction between ILs and model compounds. Later, we have utilized the concept of double salt ionic liquids (DSIL) for INA, a penta-morphic system, to investigate the variation in the polymorphic outcome. This approach helped to obtain the forms that were otherwise inaccessible in ILs.

## Abstract 2

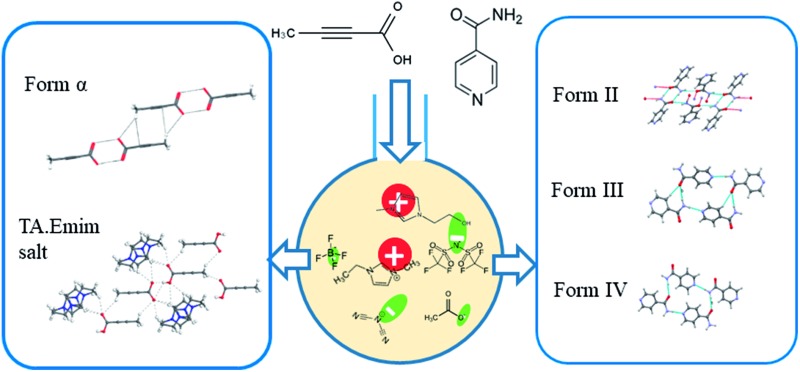
The adoption of ionic liquids as crystallization solvents for polymorphic screening opens a new window for the applications of ILs, which may allow one to access the forms difficult to crystallize from organic solvents.

## Introduction

Polymorphism in molecular solids has been of immense interest to many areas of organic solid-state chemistry.^1^,^2^ The significance of polymorphism is often realized by the different physiochemical properties exhibited by the polymorphic systems. For example, different polymorphic forms of the active pharmaceutical ingredients can have significantly different solubility and dissolution rate resulting in changes in bioavailability.^3^ Polymorphism mainly arises due to packing, conformational, or synthon differences of the interacting molecules. Packing polymorphism is often related to the late stages of crystallization to minimize the lattice energy and may be influenced by the solvents to optimize the structural organizations. On the other hand, the occurrence of conformational or synthon polymorphism can sometimes carry a solution-structure link.^4^,^5^ Between these two types, synthon polymorphism is more likely to be related to the respective aggregates in solution, as this kind of polymorphism arises from differences in primary synthons, which are also the primary recognition units. Although polymorphism in organic molecules has been studied for years, it was only until recently when systematic attempts have been made to understand this phenomenon. Crystallization of any form involves two major steps: at first, a crystal phase nucleates and in the second step, the nuclei grow into crystals of measurable size.^6^ In the pre-nucleation stage, aggregates can be formed through intermolecular interactions while in the growth stage, it is sometimes possible to deduce the fundamental units of growth, commonly known as *growth units*. As a nucleus is difficult to catch with the present set of techniques, depicting the nature of interactions present in the pre-nucleation aggregates as well as in the growth units and understanding their interrelationship often shed light on the nucleation events.^7^–^9^ Supramolecular synthons^10^ which encapsulate most of the geometrical and chemical details of the crystal structures and are often identified based on their occurrence in a handful of crystal structures, can be very useful to this end as they can exist in both the stages.

**Fig. 1:**
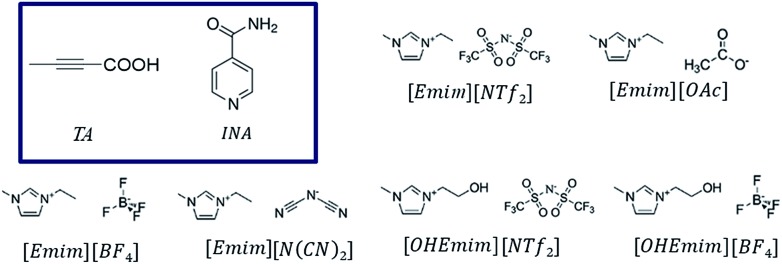
Chemical diagrams of the pure ionic liquids and the model compounds (in the box) used in this study.

While traditional solvents are often used in such studies, they offer only a limited range of intermolecular interactions. Although some of the physical properties of organic solvents can be tuned through mixing, the number of solvents and available interactions are often limited. Ionic liquids, on the other hand, can be designed and synthesized by varying a vast number of cations and anions, offering a wide liquidus range with tunability. Moreover, the primary interactions in ILs differ from those in the conventional organic solvents. While organic solvents are dominated by hydrogen bonding, dipole–dipole or dispersive interactions, ILs have the added effect of electrostatic interactions. Due to the presence of at least two types of ions, ILs possess nanoscale ordering that is absent in conventional organic solvents.^11^–^14^ This unique nature of ILs can be potentially utilized in crystallization of polymorphic systems especially to expand the polymorphic landscape of compounds mainly by tuning the ions that affect the charge ordering in the ILs. However, prior to such attempt, one needs to understand the extent of self-association of organic compounds in ionic liquids and the related crystallization mechanisms.^15^,^16^ It would also be interesting to see how the different kind of interactions in ILs affect the crystallization outcome. In addition, ILs offer tunability of solvent composition through the formation of double salt ionic liquids (DSIL).^17^,^18^ In DSILs, two or more ionic liquids are mixed in specific ratios resulting in a double salt that sometimes exhibits significantly different interactions between the components leading to different ion clusters than the ones present in parent ILs. DSILs also provide the additional advantage of extracting suitable solvent properties from multiple ionic liquids.^18^ Despite these advantages, the crystallization in ionic liquids is rarely explored mainly due to the difficulty to crystallize the material.^16^ It was only recently when it was shown that cooling crystallization can be utilized to facilitate the crystallization of organic solids from ionic liquids.^19^ In this paper, we aim to study the role of imidazolium based ionic liquids in polymorphism of certain organic systems. These ionic liquids were chosen due to the significant number of studies on their physical properties.^15^,^20^ Using these ionic liquids, we systematically investigated the crystallization behavior of tetrolic acid (TA) and isonicotinamide (INA) first by changing the cationic and anionic components. Later, we tried to utilize some of the properties of a DSIL to tune the crystallization outcome of INA, which is a penta-morphic system. The chemical structures of the ionic liquids and the compounds that were employed in this study are given in Fig. 1.

We selected these two systems because earlier studies on crystallization of these two compounds showed plausible solution-structure link in traditional organic solvents. While TA demonstrates a simpler case by sustaining two polymorphs: α form with carboxyl dimers and β form with the catemers, INA indicates the presence of five forms where form II is sustained by the dimers and the others are formed mainly by catemeric synthons. The details of the polymorphic forms for both the compounds that are relevant to this paper are provided in Table 1. Initially, we hypothesized that TA can be used mainly to understand the crystallization mechanism whereas the INA polymorphs can be tested with respect to tunability of ionic liquids. It must be noted here that crystallizations of both compounds are not straightforward. While some of the reports on crystallizations of TA in the past showed concomitant occurrence of both the forms, some of the INA polymorphs (especially form III, IV and V) suffered from low selectivity and lack of reproducibility. As ILs are known to possess ordering which is related to their unusual solvating and crystallization properties, we thought the above systems can be used as ideal probes to see (i) how the ILs affect molecular aggregation and (ii) whether the link between solution aggregation and polymorphic outcome still exists in the chosen ILs.

**Table 1:** The polymorphs of TA and INA

Compound	Form	Synthon	Space group	Stability	Crystallization solvent
TA	α	Carboxyl dimer	*P*1	Metastable at RT	Chloroform
	β	Carboxyl catemer	*P*2_1_	Stable at RT	Ethanol
INA	I	Amide catemer	*P*2_1_/*c*	Metastable at RT	Nitromethane
	II	Amide dimer	*P*2_1_/*c*	Stable at RT	Ethanol, methanol, 2-propanol
	III	Amide catemer	*Pbca*	Metastable at RT	N/A
	IV	Amide catemer	*Pc*	Metastable at RT	Nitrobenzene
	V	Amide catemer	*P*2_1_/*c*	Metastable at RT	N/A

## Results and discussion

### A. Crystallization of TA from [Emim]-based ionic liquids

TA has two polymorphic crystal forms. The α form sustains through a carboxylic dimer synthon and crystallizes in the triclinic centrosymmetric space group *P*1. The other form, β crystallizes in the monoclinic chiral space group *P*2_1_ and shows the presence of catemeric synthon.^21^,^22^ Between these two polymorphs, α was found to be metastable while β is the stable form at the room temperature. Although carboxylic acid dimers are considered more frequent than the catemers (generally 85 : 15 in occurrence), the simultaneous occurrence of these synthons for one molecular system is unusual and the basis of such polymorphic preferences in the TA may be understood if we take a detailed look at the related structures. For example, smaller acids such as formic or acetic acids adopt catemeric synthons while crystal structure of propionic acid sustains through dimer synthons.^23^ The existence of either of the synthons in two forms of TA probably indicates the subtle balance between strong (*e.g.* O–H···O based carboxyl dimers) and weak (*e.g.* C–H···O between the methyl group and carboxyl oxygen) interactions.^24^

**Fig. 2:**
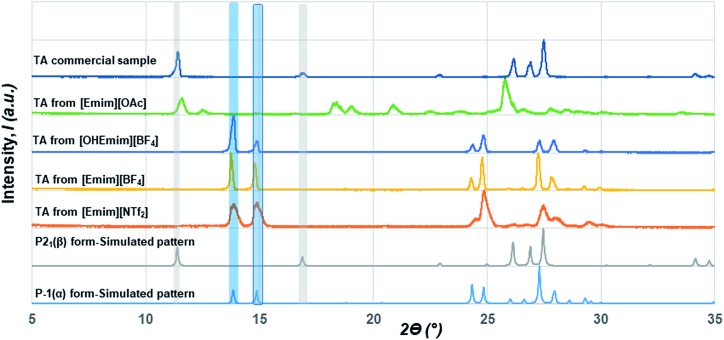
The stacked PXRD patterns showing the occurrence of α form from [Emim][NTf_2_], [Emim][BF_4_] and [OHEmim][BF_4_]. The crystallization outcome from [Emim][OAc] is different from either form α or β. All the forms obtained from the above ILs are different from the commercial sample, which consists of the β form exclusively.

Although initial studies on this compound reported a concomitant crystallization of both polymorphs from a pentane solution, a later study revealed that α form could reproducibly be obtained from chloroform (CHCl_3_) while the β form could be crystallized from ethanol (EtOH).^22^ As CHCl_3_ differs in hydrogen bond donating/accepting ability from EtOH, we chose ILs that differ in their hydrogen bond donating (HBD) or accepting (HBA) ability as well. While [Emim][NTf_2_] possesses ions that are comparable to non-polar solvents, [Emim][OAc] lies on the extreme polar end. We also employed [Emim][BF_4_] and [OHEmim][BF4] as they lie between these two extremes. Excess solutes were dissolved first at a higher temperature (60 °C) and subsequently crystallized at a lower temperature (for details, see Experimental section). The solids after being filtered under the vacuum pump were monitored by PXRD with a Cu-Kα1 X-ray source. The formation of polymorphs was monitored through the peaks around 2 = 13.8° and 14.9° for the α form and 2 = 11.4° and 16.9° for the β form. The crystallization outcome in [Emim][NTf_2_] shows the formation of the α form exclusively. The α form was also crystallized from the [Emim][BF_4_], indicating that the polarity of these solvents are close enough to give rise to similar crystallization outcome. The [OHEmim][BF_4_] was chosen to see whether the slight increase in the solvent polarity through introducing –OH group to the [Emim]^+^ cation is sufficient enough to change the crystallization outcome. However, as evident from Fig. 2, crystallization from [OHEmim][BF_4_] also resulted in the α form, indicating that slight increase in polarity through –OH substitution in the cation may not be sufficient enough to change the crystallization outcome. Solid-state FTIR spectra collected on these solids indicate the presence of carboxyl dimer around 910 cm^–1^ and therefore corroborate well with the PXRD patterns.

**Fig. 3:**
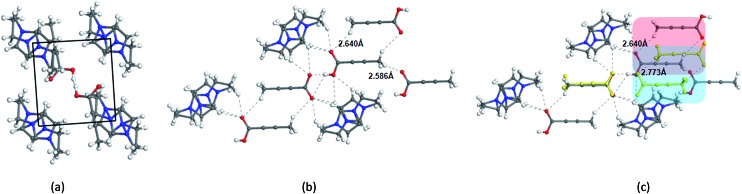
(a) Crystal packing observed in the crystal structure of [Emim][H(TA)_2_], (b) primary interactions present between the [Emim]^+^ and TA molecules. One of the symmetrically generated hydrogen was deleted in the central TA molecule to depict the interaction (c) an overlap of TA molecules in [Emim][H(TA)_2_] structure with that of the structure of the a form (yellow).

In contrast to the crystallization outcomes from non-polar solvents, while TA was subjected to crystallization from [Emim][OAc], it resulted in a different PXRD pattern. Single crystals were grown from the [Emim][OAc] by slow cooling after 3–4 days. A primary analysis of the crystal structure obtained from [Emim][OAc] reveals that the crystallization product is a salt between partially deprotonated TA and [Emim]^+^ cation, [Emim][H(TA)_2_]. Although such outcomes are not frequent, it is not surprising given the lower p*K*_a_ value of TA. The asymmetric unit contains one-half molecule of Emim and one molecule of TA. The short O···O distance between them (2.4519(18) Å) and charge balance considerations (the unit cell contains one [Emim]^+^ cation and two TA molecules) are indicative of partial deprotonation of the TA. It must be noted here that the ability of polar ILs, unlike the traditional organic solvents, to undergo such proton exchange may be used further to control the formation of polymorphs, pseudo-polymorphs, or new salts as in the case of [Emim][H(TA)_2_].^16^ The hydrogen atom on oxygen atom O1 in the TA molecule was modeled as half-occupied (Fig. 3). TA oligomeric anions interact with each other through O–H···O^–^ interactions. This catemer is surrounded by the [Emim]^+^ cations by C–H···O interaction between the imidazolium ring protons and carboxylate/carboxylic acid group. More intriguingly, these oligomeric anions, that are reminiscent of discrete catemers, propagate through the structure by weak C–H···O interactions both in and out of plane of TA/Emim. Some of these secondary interactions resemble the secondary interactions present in the α form (Fig. 3c) indicating these C–H···O interactions might carry some importance even at the stage of solution aggregation.

### B. Mechanistic investigations into the crystallization of TA from [Emim]-based ionic liquids

**Fig. 4:**
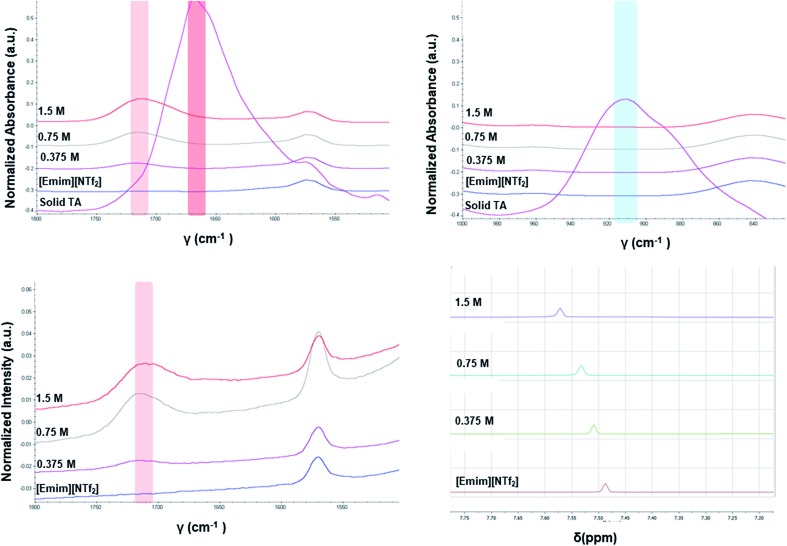
Top: FTIR spectra of TA in [Emim][NTf_2_]; bottom: Raman spectra of TA in [Emim][NTf_2_] (left), ^1^H NMR spectra of TA in [Emim][NTf_2_] (right). The solid TA as mentioned in the FTIR spectra was also crystallized from [Emim][NTf_2_].

The different crystallization outcome from polar ([Emim][OAc]) and non-polar ionic liquids (such as [Emim][NTf_2_] or [Emim][BF_4_]) indicate towards the increased interaction between polar ionic liquids and the TA. Especially, the [Emim][H(TA)_2_] as obtained from [Emim][OAc] reveals that [Emim]^+^ interacts with the TA while [OAc]^–^ might play the major role in extracting proton from one of the TA molecules.^25^ In this regard, it would be intriguing to investigate how the interaction between ILs and TA affects the formation of synthons in the solution. For example, does the polymorphic outcome from [Emim][NTf_2_] result from carboxylic acid dimers in solution as shown previously in the traditional solvents? Previous studies have shown^22^ that the solution FTIR spectra can provide insights to this end by monitoring two peaks in the fingerprint region: one around 910–930 cm^–1^ due to bending of the carboxylic acid dimer and the other around 1660–1680 cm^–1^ in carbonyl stretching region due to the hydrogen bonded carboxyl group in dimers. The FTIR spectra obtained for three different concentrations of TA in [Emim][NTf_2_] reveal two main features: (i) there is no peak around 910–930 cm^–1^ that signifies the presence of dimer. (ii) The C=O stretching frequency is around 1715 cm^–1^ which should be around 1660–1680 cm^–1^ if dimers are present in the solution. In addition to these, the solutions were also analyzed by Raman spectroscopy. The same solutions prepared for the FTIR study showed similar peak position for carbonyl stretching in Raman around 1715 cm^–1^. Due to the internal symmetry of carboxyl dimer, the symmetric carbonyl stretching is only Raman active while the asymmetric carbonyl stretching is only IR active. If dimer is formed in solution, the FTIR and Raman peak positions would differ significantly. In [Emim][NTf_2_], as shown in Fig. 4, the FTIR and Raman peaks for the carbonyl stretch appear in similar position, indicating the absence of dimers. Moreover, the positioning of the peaks at higher wave number (around 1715 cm^–1^) indicate exclusive presence of the monomers.

**Fig. 5:**
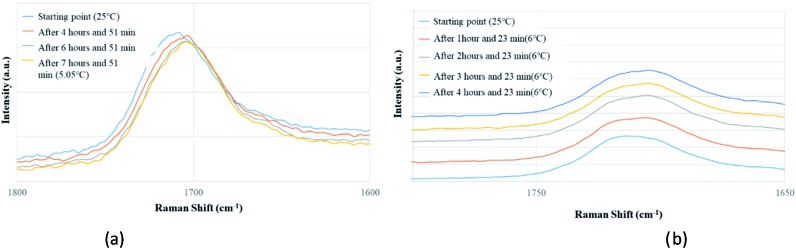
*In situ* Raman experiments of TA (a) in EmimNTf_2_ (from 25 °C to 5.05 °C), (b) in EmimBF_4_ (from 25 °C to 6 °C).

Apart from analyzing the solutions with different concentrations *via* FTIR and Raman, we also prepared a saturated solution of TA in [Emim][NTf_2_] at 25 °C and cooled it down gradually to a lower temperature (6 °C) to see whether carboxyl dimers are present while the solution approaches a supersaturation closer to nucleation. We tried to monitor the peak around 1715 cm^–1^ by *in situ* Raman spectroscopy based on the hypothesis that with the formation of dimer, this peak will either split (in case monomer and dimer both exist in solution) or shift to a significantly lower frequency (if the solution at lower temperature contains dimers exclusively). Although the peaks shift from 1712 cm^–1^ to 1705 cm^–1^ (Fig. 5a) while cooling, it clearly indicates that dimers are absent even at a lower temperature. As above evidences clearly indicate towards predominance of TA monomers in the solution, we tried to look into the role of IL in solvating TA molecules. As FTIR and Raman spectra, which are dominated by [NTf_2_]^–^ anion, showed minimal shifts in the respective peak positions for the IL, we sought for ^1^H NMR experiments on the same solutions prepared for FTIR and Raman (Fig. 4) primarily to observe the behavior of [Emim]^+^ peak positions. All the imidazolium protons shift towards downfield as compared to the pure [Emim][NTf_2_], indicating increased hydrogen bonding which might result from the interaction between TA molecules and [Emim]^+^ cations.

As all the evidences obtained from the solution studies indicate towards the existence of monomers and possible solvation by [Emim]^+^ cations, a question still persists how dimer containing α form evolved from most of the non-polar ionic liquids. In order to get further insights into this, we sought to investigate the primary growth units responsible for the crystallization of TA through attachment energy calculations (for details, see ESI†). As the polymorphic outcome from the non-polar ILs (such as [Emim][NTf_2_] or [Emim][BF_4_]) matched well with the calculated PXRD pattern of the α form, the simulated pattern was indexed to find out the major faces in the α form. A subsequent morphology calculation in Materials Studio revealed the presence of seven unique faces. All these faces were matched with the indexed faces of the calculated PXRD pattern of the α form. The unique faces in form I are: (100), (010), (01–1), (001), (1–11), (110), (1–1–1). Attachment energy calculation on these faces reveals that the attachment energy is highest for (1–11), –29.35 kcal mol^–1^ followed by (01–1) (–25.76 kcal mol^–1^) and (1–1–1) (–24.30 kcal mol^–1^).

**Fig. 6:**
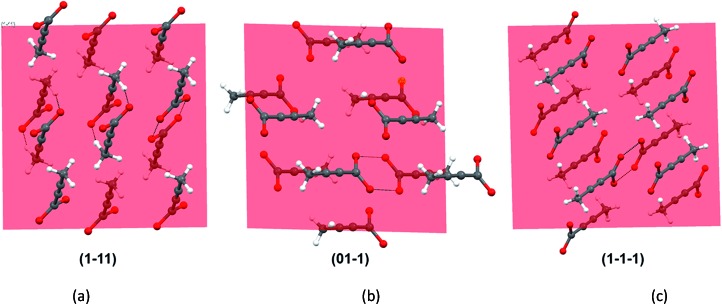
The crystal faces that contribute most to the attachment energy. Note that interactions perpendicular to (1–11) and (01–1) show significant contribution from C–H···O along with that from carboxyl dimers.

An analysis of these planes shows that the formation of C–H···O interactions are likely on the (1–11) and (01–1) planes (Fig. 6).

The FTIR and Raman studies in [Emim][NTf_2_] clearly reveal that the dimer is absent in solution even in saturated solution. The growth unit calculation on the α form of TA hints towards the formation of C–H···O to drive the crystallization to the α form. Considering the evidences obtained from the experimental and theoretical results, and insights from the crystal structure [Emim][H(TA)_2_], it seems possible that TA molecules are mostly surrounded by [Emim]^+^ cations and therefore hindered to form dimers. In such a situation, the secondary C–H···O might play a key role in bringing the molecules together during the crystallization.

### C. Crystallization of INA from [Emim]-based ionic liquids

**Fig. 7:**
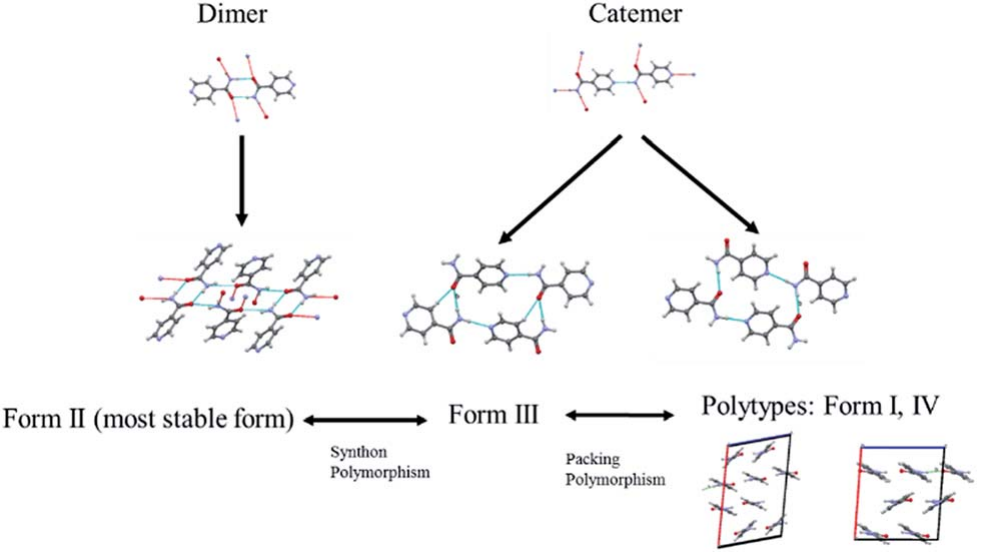
Polymorphs of INA obtained in the study and their differences.

The diversified possibilities of synthon formation and conformational flexibility of the amide group make INA a multi-polymorphic system. Among the six forms that were isolated previously, form I, II, and V crystallize in space group *P*2_1_/*c* while form III and IV crystallize in *Pbca* and *Pc* respectively.^26^–^28^ The crystal structure of form VI has not been reported so far.^29^ Form II sustains through N–H···O amide dimers and was found to be the most stable form at ambient conditions while others, sustaining through N–H···N catemers, are metastable forms under the same condition. The difference between form I and III originates from variation in secondary synthons as shown in Fig. 7 whereas form I and form IV are polytypes differing by the packing along the *b*-axis. Previous studies demonstrated that organic solvents with strong hydrogen bond donating functional groups (*i.e.* methanol, ethanol, and 1-propanol) could produce form II of INA and solvents with strong hydrogen bond accepting propensity could facilitate the formation of other metastable forms. Subsequently, form I was crystallized from nitromethane and form IV was obtained from nitrobenzene. Form III was discovered serendipitously while synthesis of a cocrystal was attempted and faced reproducibility issues ever since. Form V and form IV was obtained concomitantly as side products under cooling conditions with the presence of 3-arylbutanoic acid. This difficulty to separate form III, IV, and V under ambient conditions was mentioned by Hansen *et al.* in a previous study.^27^ Polymorphic forms of INA can be monitored *via* PXRD using Cu-Kα1 X-ray radiation. Peaks at 2*θ* = 20.8° and 23.3° represent the presence of form II while peaks at 2*θ* = 14.1°, 17.4°, and 18.4° are representative of form III. Form IV can be identified by the peaks at 2*θ* = 19.9° and 22.1°.

In light of the previous studies, we primarily considered four pure ILs, [Emim][NTf_2_], [OHEmim][NTf_2_], [Emim][BF_4_], and [Emim][N(CN)_2_] as primary crystallization solvents for INA. These selected ILs differ in their basicity and hydrogen bond donating propensity due to structural difference of constituent cation and anion. The incorporation of –OH functionality to the ethyl side chain in [OHEmim][NTf_2_] makes it a better hydrogen bond donor compared to [Emim][NTf_2_]. On the other hand, changing the anion to [BF_4_]^–^ in [Emim][BF_4_] results in slightly increased hydrogen bond accepting ability, which can be amplified further with [N(CN)_2_]^–^ in [Emim][N(CN)_2_]. The viscosities of all four ILs are lower than 60 cP at ambient temperature and the appropriate dissolving capacity makes them suitable solvents for cooling crystallization.

**Fig. 8:**
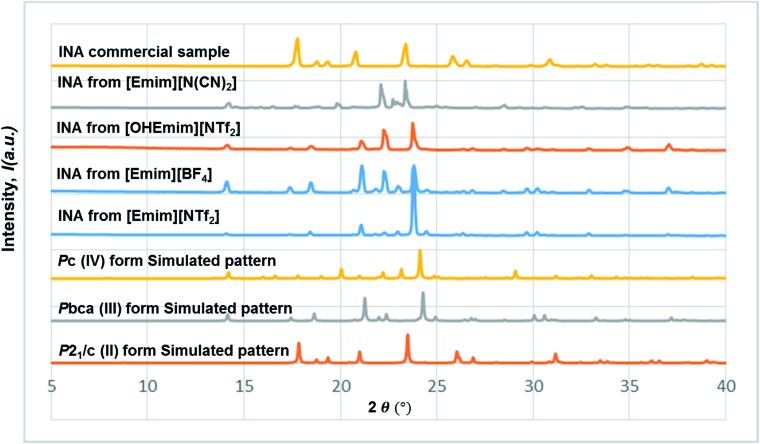
The stacked PXRD patterns indicate the occurrence of form III from [Emim][NTf_2_], [OHEmim][NTf_2_], [Emim][BF_4_], and form IV from [Emim][N(CN)_2_]. All the forms obtained from the above ILs are different from the commercial sample, which contains form II exclusively.

In all the ILs except for [Emim][N(CN)_2_], the dominant form obtained was III, a metastable form under ambient conditions. A small peak at around 2*θ* = 22.9 indicates the existence of form I (Fig. 8). In [Emim][N(CN)_2_], form IV, a form that was difficult to obtain from any organic solvents, was isolated. To summarize, it was possible to crystallize form III and IV in bulk and in a reproducible manner by employing ILs. This result demonstrates the potential of ionic liquids in polymorph screening.

However, the limited range of commercially available cationic and anionic components suitable for crystallization process may sometimes restrict the access to larger polymorphic landscape. In this case, despite the multiple structural options, we could access only two of the metastable forms by systematic variation of cations and anions in imidazolium based-ILs.

### D. Mechanistic investigations of self-association and polymorphic outcomes

A link has been observed for INA between the solute clusters and corresponding polymorphic behaviours in organic solvents.^29^ Although it was argued later that such link alone may not be sufficient to predict the polymorphic outcome, and other factors such as cooling temperature profile would also play a significant role.^27^

To determine whether such a solution-structure link exists in ILs, we investigated the structure of INA clusters in solutions *via* Raman and IR. It was noted that a Raman peak around 993 cm^–1^ is indicative of the unassociated monomers or head-to-head dimers while a peak position at 1003 cm^–1^ shows the presence of head-to-tail catemers in the solution.^29^ This is in resemblance to the Raman patterns of solid samples. To differentiate the monomers and dimers, IR spectra were also analysed for these systems, where a broad peak around 1620–1640 cm^–1^ is representative of dimers in solution. Based on the aforementioned two criteria, the results were categorized into Table 2.

**Table 2:** Solution properties and corresponding crystallization outcomes. The Raman and IR spectra in the bottom table are from [Emim][N(CN)_2_], [OHEmim][BF_4_], and [Emim][BF_4_] respectively as they represent the patterns in the described scenarios

Scenarios	Spectroscopic characteristics	Solvents for INA	Solids obtained
1-Dimers	Raman peak at 995 cm^–1^, IR peak at 1620–1640 cm^–1^	[Emim][OAC]	N/A
		[Emim][N(CN_2_)]	IV
2-Catemers and monomers	Raman peak at both 995 and 1005 cm^–1^, no distinct IR peak at 1620–1640 cm^–1^	[OHEmim][BF_4_]	N/A
		[OHEmim][NTf_2_]	III
3-Monomers only	Raman peak at both 995 cm^–1^, no IR peak at 1620–1640 cm^–1^	[Emim][BF_4_]	III
		[Emim][NTf_2_]	III

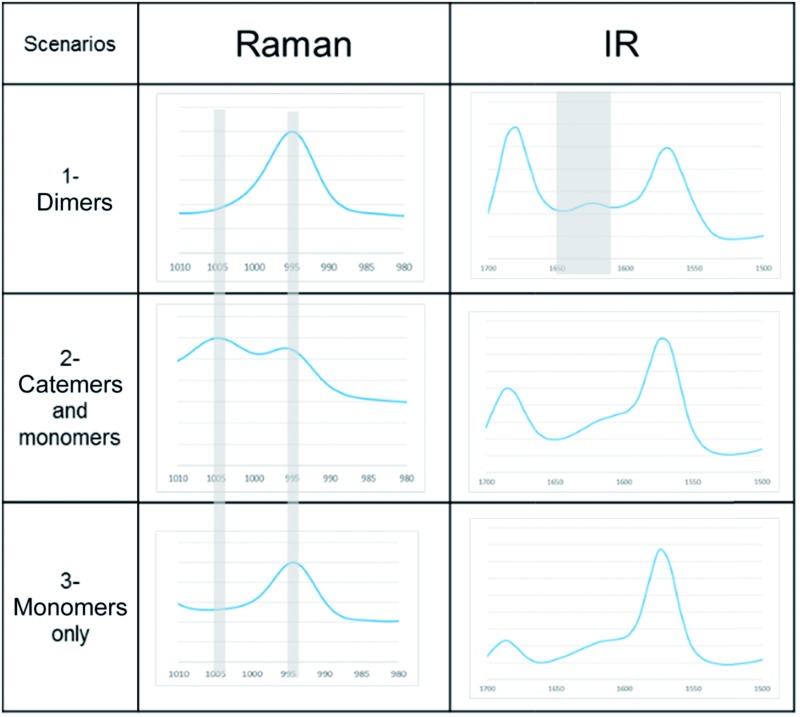

In the first scenario, IR and Raman spectra confirmed the presence of dimers in the solution when [Emim][OAc] and [Emim][N(CN)_2_] were employed as solvents. The self-associated dimers in [Emim][N(CN)_2_] contradicted with the fact that form IV, which sustains through catemeric synthon, was obtained from this IL.

**Fig. 9:**
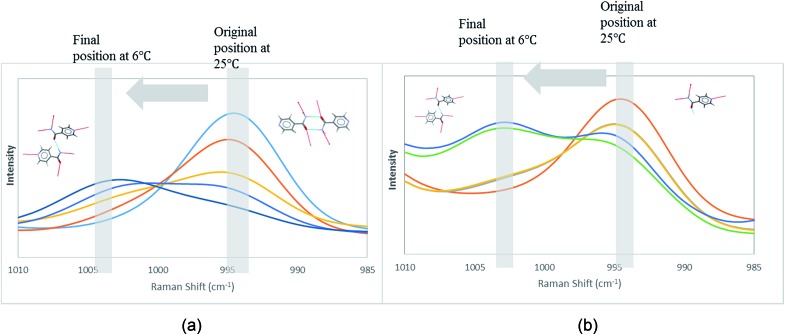
Peak transition in Raman under cooling process. (a) INA in [Emim][N(CN)_2_]. (b) INA in [Emim][BF_4_].

In order to attain better insights into such contradiction between solution aggregates and crystallization outcome, a saturated solution of INA in [Emim][N(CN)_2_] was prepared at 25 °C and cooled down to 6 °C where the nucleation event is more likely to happen. The cooling process was closely monitored *via in situ* Raman spectroscopy. Intriguingly, the original peak at 994 cm^–1^ gradually shifted to 1003 cm^–1^ as shown in Fig. 9a. A following examination under an optical microscope did not show any crystal. It is most likely that the self-association pattern of INA in [Emim][N(CN)_2_] shifted from dimers to catemers during the cooling, resulting in the formation of form IV, as observed in crystallization experiments.

In the second scenario where catemers and monomers were both present in the solution, any of the forms could be the final crystallization outcome. Catemers detected in [OHEmim][NTf_2_] corroborate well with the final polymorphic result (form III).

The possibility to detect the solution-structure link is limited in scenario 3 where no distinct peak was discovered both at 1005 cm^–1^ in Raman and at 1620–1640 cm^–1^ in IR. Monomers are the most likely to be present in this scenario due to the low solubility. NMR experiments (please check ESI† for details) conducted at different concentrations with negligible peak shift confirmed the presence of monomers. The transition from monomers (994 cm^–1^ in Raman) to catemers (1003 cm^–1^) took place during the cooling from 25 °C to 6 °C as demonstrated by the *in situ* Raman studies in [Emim][BF_4_] (Fig. 9). This corroborates well with the final crystallization outcome.

### E. DSILs to affect the polymorphic outcome of INA

As mentioned in the previous section, there have been limitations in using native ILs including restricted choice of cations and anions along with the undesirable properties (such as viscosity) of some ILs. These factors could partly restrict the accessible forms to two while [Emim] based ionic liquids were employed. For instance, the viscosity of [OHEmim][BF_4_] was too high, causing difficulty for stirring and filtering of solution. A similar example was [Emim][OAc] where the high viscosity and the strong dissolving power of the IL make the cooling crystallization at lower temperatures difficult.

In hindsight, we thought that some of these limits could be circumvented by introducing double salt ionic liquids (DSILs) which may give access to novel ionic oligomers and ion clusters. DSILs were prepared by mixing a functional IL with a base IL. A desired base IL should be chosen with lowest possible viscosity, high thermal stability and relatively non-polar properties compared to the functional ILs. [Emim][NTf_2_] has all of the three aforementioned features, thus is adopted as the base IL in forming DSIL. The DSILs were designed to modify hydrogen bond propensities of individual ILs. It should be noted that the tuning process to prepare DSILs with different hydrogen bond donating (or accepting) propensities could result in higher possibility to observe various polymorphic outcomes. Moreover, the tuning step helps to utilize functional ILs with high viscosity or superior dissolving power, therefore adds more options for solvent selection in polymorphic screening.^17^

**Fig. 10:**
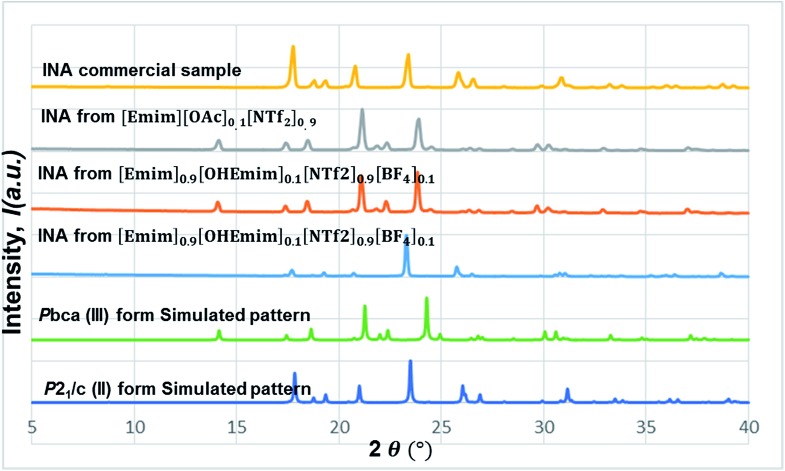
The stacked PXRD patterns indicate the occurrence of form III from [Emimv][OAc]_0.1_[NTf_2_]_0.9_. [Emim]_0.9_[OHEmim]_0.1_[NTf_2_]_0.9_[BF_4_]_0.1_ provides the possibility to see form III in some occasions and form II in others.

In this line, we first chose [Emim][OAc] to be added into the base IL at the 1 : 10 molar ratio to see whether the polymorphic outcome in this DSIL differs from what observed for [Emim][NTf_2_]. The lower proportion of [OAc]^–^ anions only influenced the ratio of minor form in the solid mixture, as shown in Fig. 8. While crystallization outcome from [Emim][NTf_2_] showed the existence of form I with form III as the dominant form, the crystallization outcome from [Emim][OAc]_0.1_[NTf_2_]_0.9_ consisted of pure form III (Fig. 10).

Tuning the hydrogen bond accepting capacity of the base IL with [OAc]^-^ anions resulted in the isolation of pure form III; attempts were also made to simultaneously tune hydrogen bond donating of cations and accepting capacity of anions. In order to achieve the tuning, we opted for [OHEmim][BF_4_] as the functional IL. The reasons for using [OHEmim][BF_4_] are two fold: (i) the high viscosity of [OHEmim][BF_4_] was unfavourable for the purpose of crystallization, yet mixing it with a low viscosity base IL would diminish the hindrance. (ii) The studies of [OHEmim][BF_4_] showed it has an acidity scale closer to alcohol.^30^,^31^ Since alcoholic solvents like methanol, ethanol, and 2-propanol induce form II of INA as final polymorphic outcome, it was intriguing to study whether mixing [OHEmim][BF_4_] with the base IL ([Emim][NTf_2_]) drives the crystallization outcome towards form II. The two ILs ([OHEmim][BF_4_] and [Emim][NTf_2_]) were mixed in 1 : 10 molar ratio, which enabled the use of –OH group from [OHEmim]^+^ with mere change of the viscosity. The cooling crystallization experiments from this DSIL led to one-third possibility of isolation of pure form II (shown in Fig. 8) and two-third possibility to obtain form III. Therefore, by employing this DSIL, the chance of obtaining a different polymorph was increased by 33% compared to the outcome from pure base IL.

## Conclusions and outlook

The investigation of crystallization of organic compounds in ionic liquids may provide one with additional polymorphic space. This study was attempted in the first place to investigate how the solution composition in the ILs relates to the polymorphic outcome and how the polymorphic space of a given molecule can be accessed with systematic changes of the IL composition. First, this study has shown that the crystallization outcome for both systems used here, in selected imidazolium-based ILs, with varying polarity and hydrogen bonding propensity, has been influenced by the interaction between ILs and the model compounds. For TA, the α form was obtained from most of the non-polar ILs while the more polar [Emim][OAc] produced an [Emim]^+^ salt of TA. In the case of INA, less polar ILs led to form III, a rare form to be observed from organic solvents in a reproducible manner. More polar [Emim][N(CN)_2_] gave access to the form IV, a metastable form often precipitated concomitantly with other forms in organic solvents. It is noted that employment of ILs as crystallization solvents helped in isolation of the forms that are difficult to obtain in organic solvents, therefore serve as an additional option for polymorphism screening.

Contrary to what was observed before in traditional organic solvents, a direct solution-structure link was absent in most of the cases. As observed in the crystal structure of [Emim][H(TA)_2_], such absence of a direct link may sometimes be attributed to the increased interaction between ionic liquids and the chosen systems. Sometimes it led to complexity: for example, INA self-aggregated into dimers in the [Emim][N(CN)_2_], yet re-oriented through cooling, as monitored by *in situ* Raman, and finally formed form IV, which sustains through catemeric synthons. It also shows that temperature may induce the change of self-association during the cooling process and should be investigated in more detail in the future. Moreover, it is worthwhile to explore how the variation of the nature of ILs helps in the detection of more robust solution-structure link. It would also be intriguing to study how the side chain length of imidazolium cations affect the polymorphic outcome.

The application of certain ILs is restricted due to their high viscosity and strong dissolving power of model compounds. This study demonstrates that the concept of DSIL can be explored to give access to more anion selections and functional groups in the multiple ILs. Intriguingly, both the DSILs discussed in this paper helped in rational tuning of the respective polymorphic outcomes. [Emim]_0.9_[OHEmim]_0.1_[NTf_2_]_0.9_[BF_4_]_0.1_ was designed as a capable hydrogen bond donor and it led to the isolation of form II which was otherwise not accessible directly from native ILs. The adoption of DSIL in this case opens a new window for the applications of ILs in general which may allow one to access more forms in polymorph screening in the future.

## Experimental details

### A. Materials

Isonicotinamide (INA), tetrolic acid (TA) were purchased from Sigma Aldrich and used as received. 1-Ethyl-3-methylimidazolium bis(trifluoromethanesulfonyl)imide ([Emim][NTf_2_]), 1-ethyl-3-methylimidazolium dicyanamide ([Emim][N(CN)_2_]), 1-ethyl-3-methylimidazolium acetate ([Emim][OAC]), 1-ethyl-3-methylimidazolium tetrafluoroborate ([Emimv][BF_4_]), 1-(2-hydroxyethyl)-3-methylimidazolium bis(trifluoromethanesulfonyl)imide ([OHEmim][NTf_2_]), and 1-(2-hydroxyethyl)-3-methylimidazolium tetrafluoroborate ([OHEmim][BF_4_]) were purchased from Iolitec and stored in a dry-box under a nitrogen atmosphere. The amount of water in these ionic liquids was negligible. However, water content was calculated for each ionic liquid to get a quantitative estimate. For details, please see ESI.†

### B. Preparation of double salt ionic liquids (DSILs)

The preparation and storage of DSILs were carried out in nitrogen box to avoid the adsorption of water moisture. The mass of each IL component was calculated according to the target molar ratio of cations and anions in DSIL. Constituent ILs were weighted and mixed together *via* a vortex mixer.

### C. Crystallization from ILs and DSILs

The crystallization experiments of TA were performed in Avantium Crystal16™ by adding known amounts of the compound in the respective IL and heating it up to 60 °C. The dissolution was monitored *via* %transmission implemented in Avantium Crystal16™. After equilibrating at the 60 °C the solution was further cooled down to 5 °C at the rate of 0.5 °C min^–1^. The crystallization product obtained was filtered under vacuum prior to further analysis. For the crystallization of INA, saturated solution of model compounds in ILs or DSILs was prepared under 60 °C and rapidly passed through a 0.2 μm PTFE membrane filter. Approximately 1 mL of the obtained clear solution was then transferred to VWR® Screw-Thread vial. For the control of cooling rate, the vials were loaded into an Avantium Crystal16™ and cooled from 60 °C to 5 °C at a rate of 5 °C min^–1^.

### D. Powder X-ray diffraction (PXRD)

Solid samples were analyzed using a PANalytical X'Pert Pro diffractometer at 45 kV with an anode current of 40 mA. Solid samples were loaded flat on a zero background sample holder without intensive grinding to prevent polymorphic transformation. PXRD data collection was done in reflection mode after placing the samples on a spinner stage. Data were collected over the range 2 = 5–40° (or 5–35°) with a Cu K_α1_ source (*λ* = 0.154056 nm).

### E. Single crystal X-ray diffraction (SCXRD)

Single-crystal X-ray diffraction data (φ and ω scans) were collected at 100 K on a Bruker-AXS X8 Kappa diffractometer coupled to a Bruker APEX2 CCD detector using Mo Kα radiation (*λ* = 0.71073 Å) from an IμS microsource. Data reduction was carried out with the program SAINT^32^ and semi-empirical absorption correction based on equivalents was performed with the program SADABS^33^ The structure was solved with dual-space methods using the program SHELXT^34^ and refined and refined against *F*^2^ on all data with SHELXL-2014 (ref. 35) using established refinement strategies.^36^ The hydrogen atom on carboxylic oxygen of TA is shared between the two oxygen atoms on the TA dimer; however two discrete sites are found for it (coordinates for the hydrogen atom in question were taken from the difference Fourier synthesis), even though only one of those two sites is crystallographically independent. The half Emim ion is located on a crystallographic inversion center and, since Emim is not inversion symmetric, is disordered accordingly. Application of the inversion symmetry generates the second half of the molecule, which overlaps, with the first half in a different orientation. A crystallographic table and a ORTEP diagram for this structure are provided in the ESI.†

### F. FTIR and Raman spectroscopy

Solution sample analysis was performed under an IdentifyIR® FT-IR Spectrometer and *via* a Raman Workstation from Kaiser Optical Systems Inc. on a glass slide wrapped by aluminum foil. *In situ* monitoring was carried out using Raman Rxn2Hybrid (Kaiser Optical Systems Inc). Appropriate amount of sample solution was placed in a metal container and experiments were performed without stirring. This setup was attached to the temperature control unit and monitored with a 785 nm Raman sampling probe.

### G. NMR spectroscopy

NMR spectra were collected in Bruker-400 MHz instrument. Solubility experiments were performed by diluting saturated solutions with deuterated acetone. The other experiments were performed in a co-axial way by keeping the IL solution in a glass capillary and using deuterated acetone as lock. All the experiments were performed at room temperature.

### H. Materials studio calculations

The experimental patterns were collected for the samples and matched with the simulated patterns obtained from the single crystal structure already in the Cambridge Structural Database (CSD).^37^ When there was a good match in the peak positions, simulated pattern was taken for the face indexing and subsequently indexed by the Reflex module already implemented in Materials studio. As all the unique faces present in the simulated pattern are observed in the experimental patterns, attachment energy calculations were performed in the Morphology module of Materials Studio by taking the crystal structure of the α form as input.

## Conflicts of interest

The authors declare no conflicts of interest.

## Supplementary Material

Supplementary information

Crystal structure data
