# Supplementary material for: Exploring the role of ionic liquids to tune the polymorphic outcome of organic compounds
Source: Chem Sci. 2017 Dec 22;9(6):1510–20. doi: 10.1039/c7sc04353h (PMC5887107; doi:10.1039/c7sc04353h)
Supplement: Supplementary file 1 [file SC-009-C7SC04353H-s001.pdf]

Electronic Supplementary Material (ESI) for Chemical Science.
This journal is © The Royal Society of Chemistry 2017

Exploring the role of ionic liquids to tune the polymorphic outcome of organic compounds

Qingying Zeng†‡, Arijit Mukherjee†‡, Peter Muller^, Robin D. Rogers≠, Allan S. Myerson†*

† Novartis-MIT Center for Continuous Manufacturing and Department of Chemical Engineering Massachusetts Institute of Technology, 77 Massachusetts Avenue, Cambridge, Massachusetts 02139 (USA)

≠ Department of Chemistry, McGill University 801 Sherbrooke St. W., Montreal, QC H3A 0B8 (Canada)

^Department of Chemistry, Massachusetts Institute of Technology, 6-331, 77 Massachusetts Avenue, Cambridge, Massachusetts 02139, United States

‡ These authors contributed equally to the manuscript.

*Corresponding author email: myerson@mit.edu

Supporting Information

Contents

Index	Title	Page number
S1	Comparison of Tetrolic acid polymorphs with other similar systems	2
S2	Crystallographic information table for [Emim][H(TA)$_2$]	2
S3	ORTEP diagram for [Emim][H(TA)$_2$]	3
S4	Attachment energy calculations on α-dimer of TA	3
S5	Raman spectra of Isonicotinamide (INA) catemer (peak at 1003cm^{-1}) form and dimer form (peak at 995cm^{-1})	4
S6	Simultaneous examination of Solid and IL Raman spectra (IL in the example: [Emim][BF$_4$])	4
S7	NMR spectra showing INA monomers in [Emim][NTf$_2$], [Emim][BF$_4$]	5
S8	Water content calculation in the used ILs	6

S1. Crystal structures of two tetrolic acid polymorphs

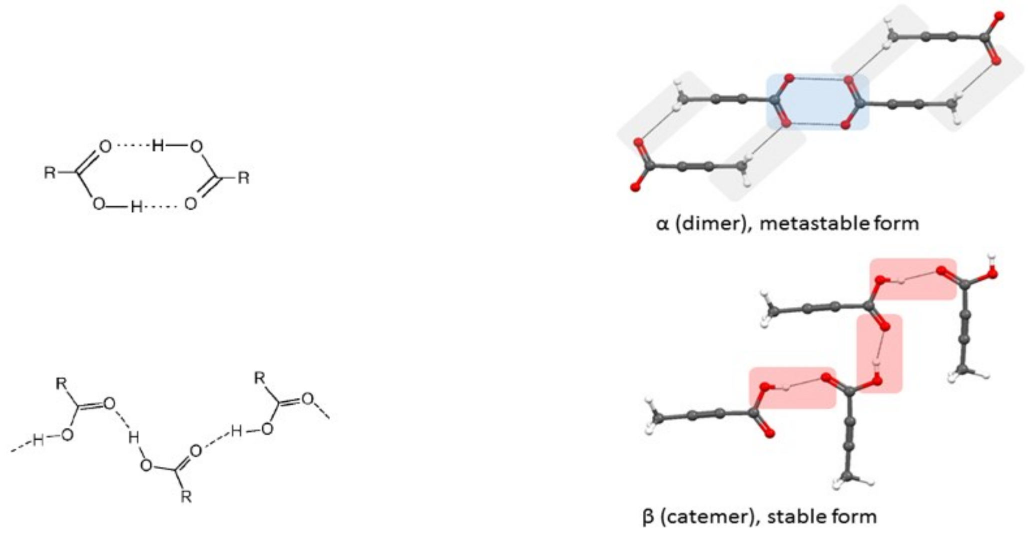

(a) (b)

Figure S1. (a) Possible synthons in the polymorphs of tetrolic acid, (b) α form shows the presence of carboxyl acid dimer whereas the β form sustains through the catemer synthon.

S2. Crystallographic details for [Emim][H(TA)$_2$]

Name	1-Ehtyl-3-methylimidazolium tetrolate
Formula	$C_{14}H_{18}N_2O_4$
Molecular weight	278.30
Crystal system	triclinic
Space group	P -1
a(Å)	6.746(3)
b(Å)	7.246(3)
c(Å)	7.434(3)
α(º)	77.801(7)
β (º)	83.650(8)
γ(º)	88.409(7)
Volume (Å^3)	353.0(2)
Z	1
$\rho_{calc(}$g/cm^3)	1.309
F(000)	148
Crystal size (mm)	0.11 x 0.22 x 0.41
μ (Mo Kα) (mm^{-1})	0.097
Temp (K)	100(2)
Θ range for data collection	2.8, 33.6
R_1	0.0624
wR_2	0.2064
Goodness of fit	1.08

Reflns collected	2771
Unique reflns	2771
Observed reflns	2101
CCDC no.	1577981

S3. ORTEP diagram of [Emim][H(TA)$_2$]

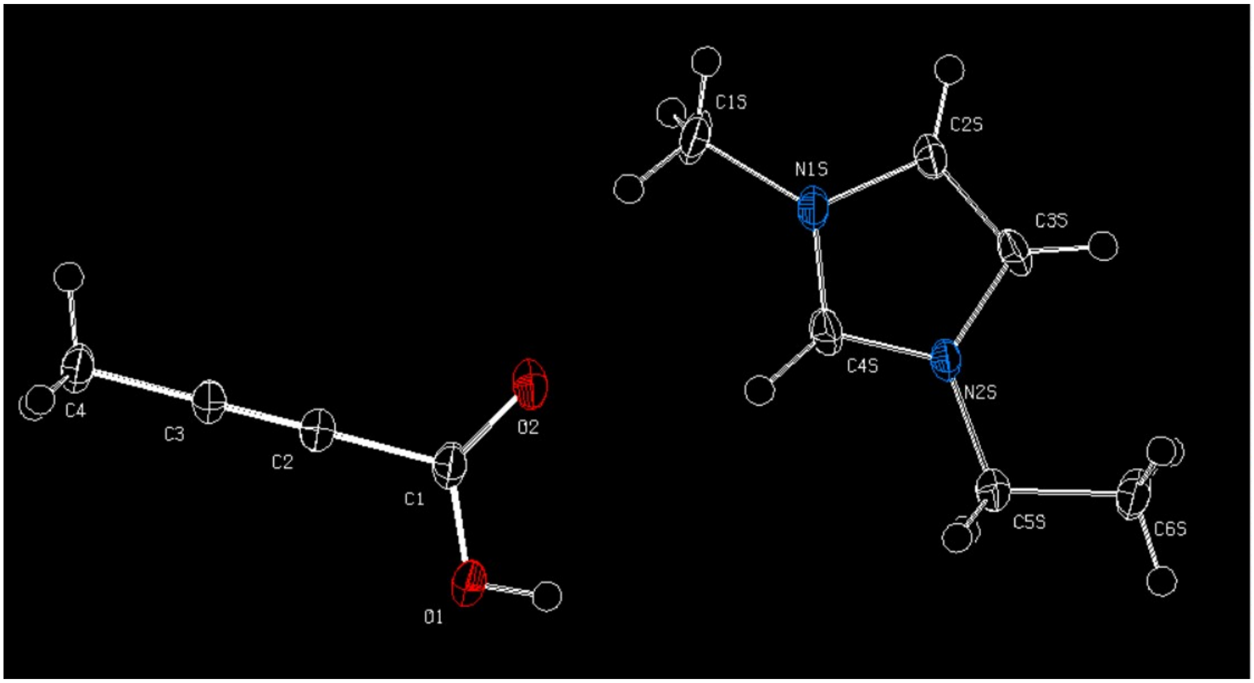

S4. Attachment energy calculations on α-dimer of TA

Attachment energies were calculated in the Morphology module of the Materials Studio. The attachment energies for the seven unique faces of the α-dimer of TA are given in the following table.

**Faces**	**E_{att}(Kcal/mol)**	**Eatt (vdW)**	**Eatt(electrostatic)**
**(100)**	-18.18	-14.29	-3.89
**(010)**	-14.76	-13.19	-1.57
**(01-1)**	-25.76	-10.36	-15.41
**(001)**	-8.99	-8.5	-0.49
**(1-11)**	-29.35	-13.92	-15.42
**(110)**	-16.94	-16.44	-0.50
**(1-1-1)**	-24.30	-9.40	-14.90

S5. Raman spectra of Isonicotinamide (INA) catemer (peak at 1003cm^{-1}) form and dimer form (peak at 995cm^{-1})

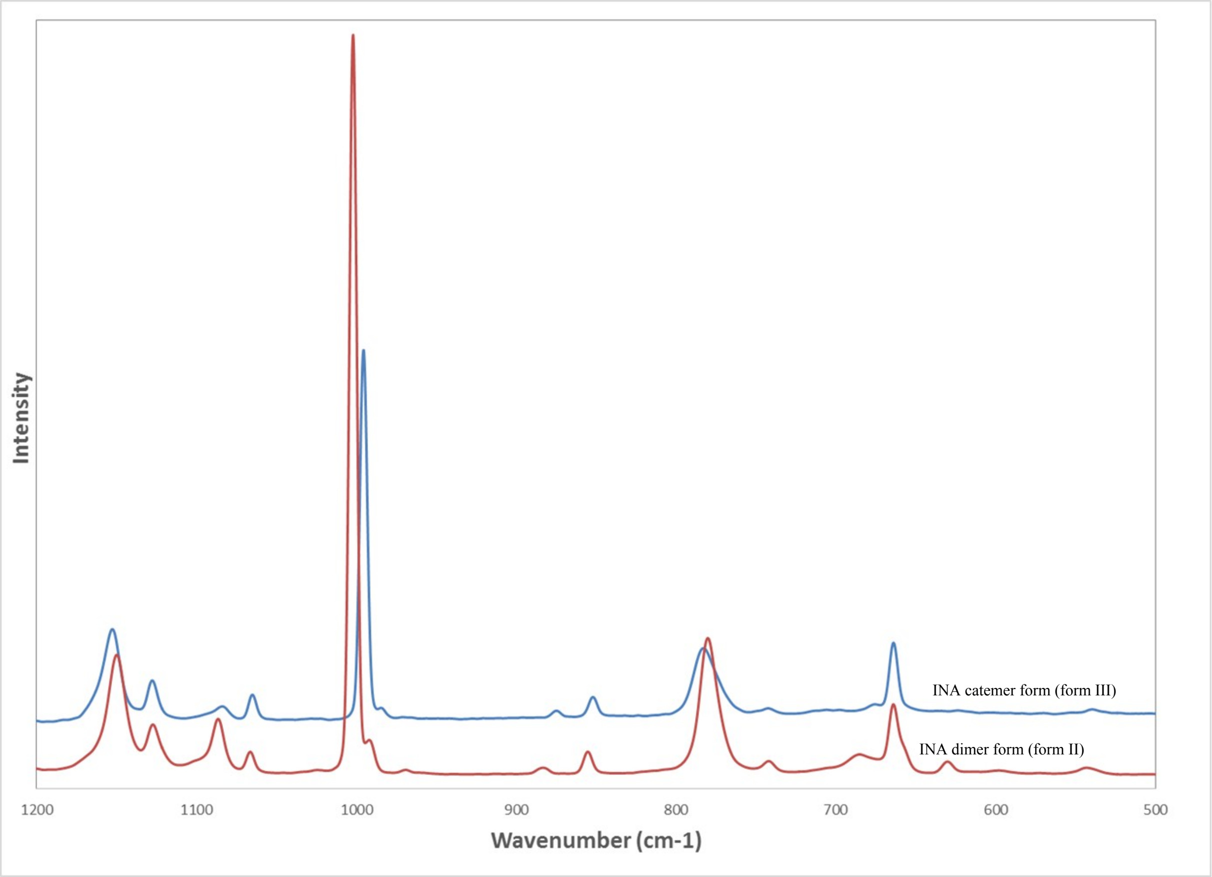

S6. Simultaneous examination of Solid and IL Raman spectra (IL in the example: [Emim][BF_4])

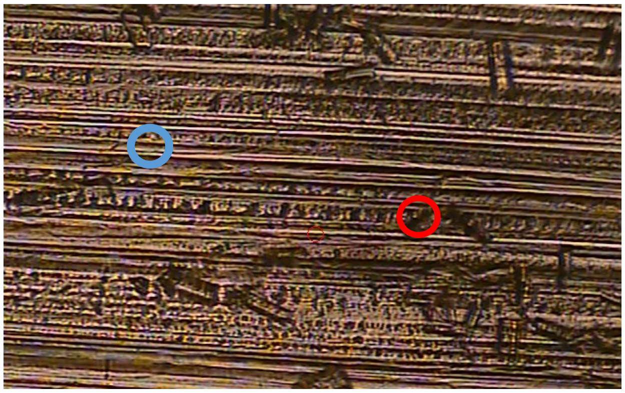

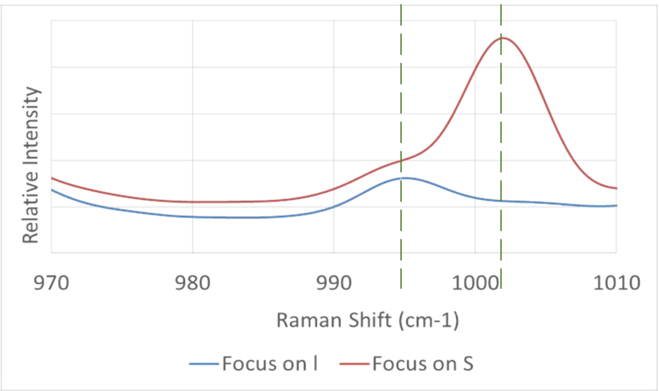

S7. NMR spectra showing INA monomers in [Emim][NTf_2], [Emim][BF_4]

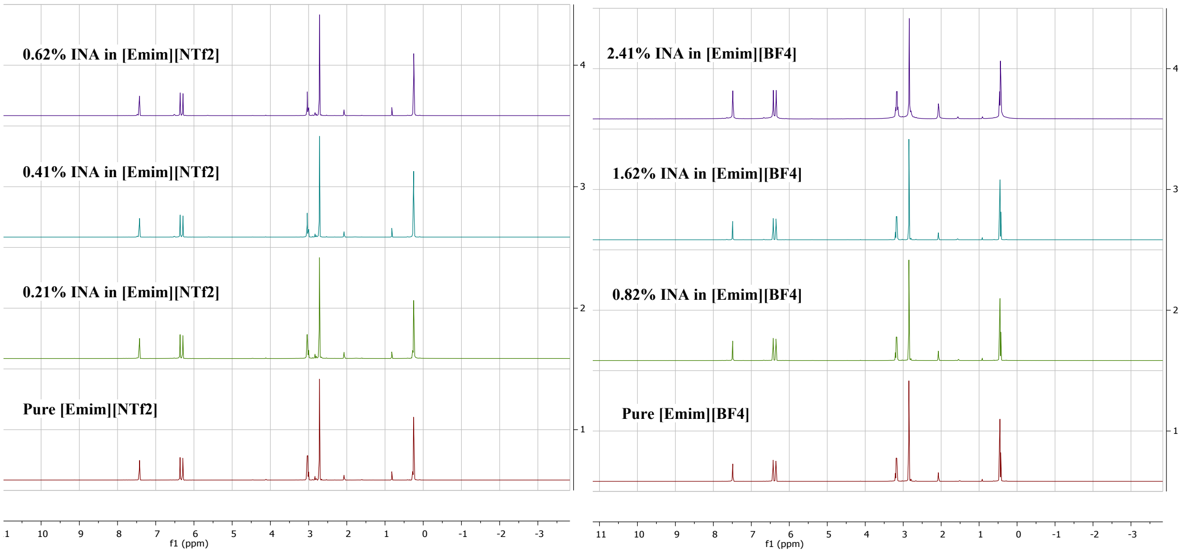

2H chemical shifts of [Emim] in ppm

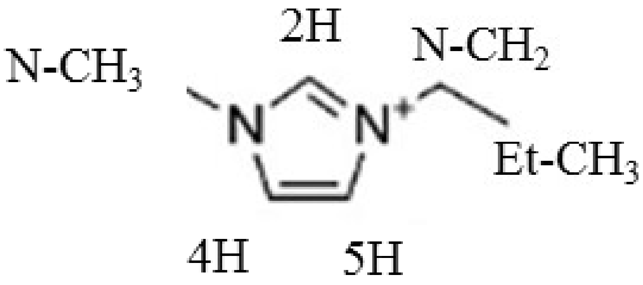

Sample	2H	4H	5H	N-CH_3	N-CH_2	Et-CH_3
[Emim][NTf_2]	7.49	6.43	6.35	3.19	2.85	0.45
Low	7.49	6.43	6.35	3.19	2.85	0.45
high	7.49	6.43	6.35	3.18	2.85	0.45
Saturated	7.49	6.42	6.35	3.18	2.84	0.44
[Emim][BF_4]	7.43	6.36	6.29	3.04	2.72	0.25
low	7.43	6.36	6.29	3.04	2.72	0.25
high	7.43	6.36	6.29	3.03	2.72	0.25
Saturated	7.43	6.36	6.29	3.03	2.72	0.25

S8. Water content calculation in the used ILs

Ionic Liquid Name	Measurement 1 (ppm)	Measurement 2 (ppm)	Measurement 3 (ppm)	Water Content (ppm)	Water Content (%)
[Emim][NTf_2]	260	262	282	268±12	0.03
[Emim][OAc]	4524	4405	4261	4397±132	0.44
[Emim][$N(CN)_2$]	1595	1711	1793	1700±99	0.17
[OHEmim][BF_4]	1740	1732	1853	1775±68	0.18
[OHEmim][NTf_2]	268	293	284	282±13	0.03
[Emim][BF_4]	1030	1014	1011	1018±10	0.1
